# The Promise of Mindfulness‐Based Interventions: A Stress‐Reduction Strategy for Testicular Cancer Survivors' Health‐Related Quality of Life

**DOI:** 10.1002/cam4.71523

**Published:** 2026-02-06

**Authors:** Michael J. Rovito, Colin F. O'Mahony, Kamalie Thomas, Keith Brazendale, Ciaran M. Fairman

**Affiliations:** ^1^ University of Central Florida Orlando Florida USA; ^2^ University of South Carolina Columbia South Carolina USA

**Keywords:** cancer survivorship, health‐related quality of life, mindfulness techniques, testicular cancer

## Abstract

**Background:**

Testicular cancer (TC) disproportionately affects younger men and carries unique psychosocial and physiological consequences that extend well beyond treatment. Despite favorable survival rates, TC survivors frequently report diminished health‐related quality of life (HRQoL). These burdens are often compounded by masculinity‐related identity disruptions, fear of recurrence, sexual dysfunction, and a lack of tailored psychosocial support. Existing interventions remain limited, with most programs focused on physical rehabilitation or early detection. Few address the multidimensional stressors that shape the TC survivorship experience.

**Aims:**

This narrative review examines the literature on HRQoL among TC survivors and evaluates the potential of mindfulness‐based interventions (MBIs) as a viable therapeutic strategy.

**Materials and Methods:**

A comprehensive literature search identified artivles that were produced in the past two decades+ that investigated the relationship between MBIs and TC survivor HRQoL.

**Discussion:**

Evidence from broader cancer populations demonstrates that MBIs, such as meditation, breathing exercises, and mindfulness‐based psychoeducation, can reduce psychological distress and promote emotional regulation. Programs like MindCAN have shown promise in improving self‐awareness, affect, and coping. Importantly, MBIs offer a low‐cost, flexible, and sustainable approach that aligns with the autonomy often valued by men navigating survivorship.

**Conclusion:**

To date, no fully developed mindfulness trials have been developed specifically for TC survivors. Given the early age of diagnosis and long survivorship trajectories, as well as the unique psychological and physiological health outcomes associated with TC, MBIs may be especially well‐suited to this population. This review calls for a renewed focus on implementing mindfulness‐based strategies designed for the lived realities of TC survivors. Doing so may meaningfully enhance post‐treatment outcomes, reduce disparities in male mental health care, and promote holistic wellness in one of the most underserved cancer survivor populations.

## 1 Background

### 1.1 The Stress of Cancer

The current literature suggests demanding psychosocial and physiological effects upon a person undergoing cancer diagnosis, treatment, and/or survivorship [1, 2]. The experience of cancer at any phase can evoke physical and emotional highs and lows [3, 4]. These floor and ceiling experiences may not be limited to solely the patients' experience, as research shows that family members and primary caregivers are also impacted by their experience with cancer [5, 6].

Cancer survivors report that psychosocial stressors, such as fear of recurrence, are a top concern and listed as one of their primary unmet needs [7]. Such psychological stressors are linked with anxiety, depression, poorer health‐related quality of life (HRQoL), compliance with follow‐up, among other health issues [7, 8, 9, 10]. Lewandowska et al. [11] indicated that out of over 500 sampled cancer survivors, 80 + % had a negative experience related to their cancer journey experience. These authors more specifically found that cancer survivors suffered from anxiety (61%), had depressive symptoms (58%), and/or experienced anger (33%) because of their experiences, with isolation serving as a primary catalyst for negative mental health outcomes.

Taniguchi & Mizuno [12] discovered an increased level of stress among patients with postsurgical cancer as compared to the general population. The article highlighted that increased stress levels were also moderately correlated with perceived illness‐related demands [12]. Langford et al. [13] further reported that out of nearly 1000 patients undergoing chemotherapy for breast, gastrointestinal, gynecological, or lung cancer, ~40% were “stressed” as compared to “normative” (~54%) and “resilient” (~6%). Out of the “stressed” group, more than 30% were classified as having at least partial PTSD, if not a fully diagnosed case.

Swartzman et al. [14] additionally indicated that cancer survivors had just shy of a 70% increased chance of having PTSD than the matched controls. These authors extended that argument by reporting that not only are the cancer survivors themselves at higher risk for PTSD, but also their caregivers, family, or others close to the patient. The study emphasized that survivors of adolescent and young adult cancer were more than twice as likely to be diagnosed with a psychological disorder, such as depression, anxiety, and cancer worry.

The multidimensionality of one's experience with cancer can be a *total* one, meaning that one's mental, physical, spiritual, social, environmental, financial, and professional worlds can be significantly affected in the short‐ and long term. Therefore, it is important for healthcare practitioners to administer a *total* mode of treatment mindset or at least be open to exploring alternative and complementary options for recovery. Previous research has shown that a multidimensional approach to treatment and recovery can improve coping and participation among cancer survivors [15]. Li et al. [16], for example, discussed the impact of the cancer survivorship process on social health. The authors found that young adult cancer survivors who experience symptoms of depression and anxiety were associated with having high levels of social isolation.

Financial toxicity was also reported to negatively impact a survivor's personal and professional life more so compared to adults with no cancer diagnosis [1, 2]. Examples of the multidimensionality of the cancer survivorship journey are numerous, but pale in comparison with the seemingly primary focus on acute physicality.

### 1.2 Stress Among Testicular Cancer Survivors

Testicular cancer (TC) survivors are at an increased risk for numerous adverse health outcomes because of the disease's unique pathophysiology and treatment [16]. These individuals have increased risks for a myriad of physical and mental health concerns, not just in comparison with the general cancer‐free population, but to other cancer survivors as well [17, 18].

TC survivors are subject to mental (e.g., post‐traumatic stress disorder, depression, anxiety, and fear of recurrence) and physical health concerns (e.g., hormonal imbalances, impotence, fatigue, and overweight/obesity) [19, 20], as well as other factors (e.g., financial toxicity [21]) that adversely affect their overall sense of wellbeing from diagnosis to decades post‐treatment. Pertaining to orchiectomy cases, there are documented mental and social health implications for testicular removal due to the centrality of masculinity [22]. Alarmingly, at least 20% of TC survivors need psychological support up to 10+ years after initial treatment [23, 24]. Unfortunately, there is a dearth of information on robust, multidimensional interventions aimed at ameliorating the burden within this population. Again, the field is rife with a focus on acute physical outcomes and lacking in more long‐term recovery.

The two greatest reported psychological challenges for TC survivors are stress and anxiety, which primarily, but not exclusively, stem from sexual dysfunction, fertility, and mortality concerns [9, 23, 25]. Distress levels among TC survivors are higher than for most other cancers [23] and are even more pronounced among males who are younger, under/unemployed, less educated, and/or single. Further research indicates that almost one‐third of TC survivors report substantial psychological distress up to 12 years post‐treatment [9]. As many psychological stressors (e.g., fear of recurrence) stem from an uncertainty of the disease (i.e., lowered awareness to, and knowledge of, the health concern) [26], there is an urgent public health equity concern with TC as many males are limited in their awareness and knowledge of the disease [27]. It would be an understatement to suggest that stress, again, particularly long term, is under‐researched within the field of TC survivorship.

Previous literature suggesting the disproportionate psychological burdens experienced by survivors of TC, as well as current gaps in the literature focusing on ameliorating stress within survivors of TC, posits the importance of designing interventions that can improve psychosocial outcomes among this population. Through this review, the potential of utilizing mindfulness‐based interventions (MBI) to improve the psychological distress experienced by the TC survivorship community is discussed. The objective of this article is to review the current literature describing interventions aimed at improving HRQoL outcomes within TC survivors, as well as literature describing the impacts of MBIs within other populations.

## 2 Methods

A systematic search of the literature was conducted using the PubMed‐MEDLINE database and Google Scholar. Two searches were conducted for (a) previous interventions utilized among TC survivors that targeted psychological and psychosocial outcomes, and for (b) previous MBI interventions utilized by similar populations. For search (a), key search terms included as follows: “testicular cancer,” “survivorship,” “health‐related quality of life OR quality of life,” “stress OR distress,” “anxiety,” and “intervention OR experiment.” For search (b), key search terms included as follows: “mindfulness,” “mindfulness‐based intervention,” “MBI,” “health‐related quality of life OR quality of life,” “stress OR distress,” and “anxiety.” This included review, meta‐analytic, and experimental studies.

For search (a), interventions targeting improvements in outcomes not related to psychological health or HRQoL, as well as interventions targeting populations other than TC survivors were excluded. For search (b), interventions not grounded in mindfulness practice, as well as interventions not targeting psychological health outcomes and/or HRQoL were excluded.

## 3 Discussion

### 3.1 Interventions for TC Survivors

Much of the TC‐related behavioral intervention research produced in the past 20+ years has focused on raising awareness and knowledge about the disease among asymptomatic, disease‐free males [28, 29]. A large share of treatment‐based clinical trials that exist test the efficacy of chemotherapy and/or radiation therapy as the primary outcome [30, 31]. Relatively few interventions concentrate exclusively upon HRQoL post‐treatment grounded in health behavior theory.

One promising area of work involves physical activity programs. Some emerging evidence indicates that physical activity can improve TC survivor HRQoL and decrease depressive symptoms, anxiety, and stress levels [32]. These programs can also improve inclinations to exercise more frequently [20, 32, 33]. Outside of physical activity, Hoyt et al. [34] present the only existing behavioral therapy intervention targeted at improving quality of life among TC survivors. The authors of this study noted that survivors who engaged in goal‐focused emotion‐regulation therapy were associated with greater reductions in depressive and anxiety symptoms when compared to those in an active control group.

This previous point begs several questions: *Where should the field go?* And *What programs work to promote multidimensional health outcomes within TC survivorship*? These questions, admittedly, are not new; they are more reoccurring and ubiquitous. The field lacks focused evidence‐based guidance to address many of the reported long‐term concerns, particularly with mental and emotional wellbeing in the field of TC survivorship. Despite the (aforementioned) best efforts, more studies on stress reduction among TC survivors are needed. Even more importantly, there needs to be a diversified approach to stress reduction within this population. The centrality of mental health concerns among this population demands diverse mitigation strategies.

### 3.2 The Potential of Mindfulness‐Based Interventions

Currently, no randomized controlled trials of mindfulness‐based interventions have been conducted specifically with TC survivors. The evidence reviewed in this section therefore draws primarily from studies of breast cancer survivors, mixed‐cancer cohorts, and adolescent and young adult (AYA) populations. These groups serve as relevant comparators given shared characteristics with TC survivors: young age at diagnosis, extended survivorship trajectories, life‐stage disruptions affecting identity and relationships, and elevated rates of psychological distress. Throughout this section, we specify the populations from which findings are derived and note where TC‐specific adaptation or evaluation may be warranted.

Research shows that the human body often proves to be resilient throughout one's experience with cancer. Decat Bergerot & Cavalcanti Ferreira de Araujo [35] analyzed HRQoL among 200 patients with cancer undergoing chemotherapy at three different intervals: the first day, halfway through treatment, and the last day. The authors underscored that during the cancer treatment process, high levels of stress were observed at initial treatment but lessened over the course of their therapy regimen. This is indicative that HRQoL scores rose over the same period [35]. This showcases resilience among the patients to thrive, even if it is of their own accord without assistance.

Consistent quality assistance with any post‐treatment health concern, particularly mental health, however, is grossly inadequate. Ferrari et al. [36] outlined that although their patient sample had lower self‐reported anxiety and depression (~27% and 17.5%, respectively), approximately one in three patients reported having insufficient access to support group services. This remains to be a significant concern throughout the literature [37, 38]. So, again, back to the central question: *What can the field delve into that can provide TC survivors the mechanism to offer palliative methods for their HRQoL concerns, yet allow for the autonomy and independence that may be required of the patient to implement the treatment in lieu of a lack of support services within the healthcare system?*

New and improved methods are emerging within other disciplines and are having a significant impact. One area of research and outreach that shows promise in positively impacting cancer survivor HRQoL is the implementation of MBIs. Mindfulness meditation consists of a non‐judgmental mind space in which a person can fully engage in the present instead of focusing on the past and future (Sercekman [39]). Engagement through mindfulness can strengthen one's connection to reality and allow oneself to focus on present emotions and experiences, which can in turn reduce levels of stress and anxiety [40, 41, 42, 43]. Overall, research suggests that a significant relationship exists between mindfulness interventions and improvement in psychosocial variables among cancer survivors. Bower et al. reported that breast cancer survivors who received regular education over mindfulness practices and instruction for at‐home exercises saw a reduction in stress, depressive symptoms, fatigue, among other factors. These patients further reported improvements in the sense of meaning, peace, and positive affect.

Boyle et al. [40] indicated that the incorporation of mindfulness, kindness of others, as well as a focused effort to reduce rumination into the daily practice of breast cancer survivors reduced symptoms of depression and stress. The authors of this study reported a 3.49 reduction in mean perceived stress score from pre‐ to post‐intervention among those engaging in mindfulness awareness practices, compared with a 0.35 increase in mean perceived stress score among the control group over the same time frame.

Adding to this emerging narrative, Victorson et al. [44] examined the effects of MBIs on outcomes of stress, anxiety, and depression, and found a medium effect size of MBIs on stress (Hedges g = −0.47, *p* < 0.01), and a small effect size of MBIs on anxiety (Hedges g = −0.21, *p* = 0.02) and depression (Hedges g = −0.25, *p* < 0.01). Pedro et al. [45] corroborates this evidence in a similar meta‐analysis. The authors of this study found beneficial effects of MBIs on symptoms of anxiety, depression, and stress, as well as on physiological outcomes, such as immune‐related biomarkers [45]. This transcends the idea that MBIs may only benefit psychosocial outcomes, adding that MBIs may also have a role in improving physiological outcomes associated with cancer survivorship.

Regarding MBIs in the literature, there is one specific program worthy of an expanded summary. Goei, Lopez, and Klanin‐Yobas (2021) [46] outline the mindfulness‐based psychoeducation for cancer survivors (MindCAN) program that aimed to manage cancer survivors' physical and psychological symptoms. The eight weekly 90‐min MindCAN sessions offer two components: education and mindfulness practice. MindCAN program participants reported increased self‐awareness, pleasant body experiences, positive thinking and emotions, and relaxation. An overall improvement in HRQoL was observed among those who took part in mindfulness‐based programming. The results of the MindCAN program posit the promise of MBIs among TC survivors.

Admittedly, the effectiveness of each mindfulness‐based trial varies. Mindfulness practices, such as yoga, deep breathing, and meditation, may all yield different levels of success when used by different cancer survivor populations. This fact underscores the importance of further research to be able to conclusively understand the most effective treatments for TC survivors. For example, a growing body of literature supports MBIs as beneficial for many cancer survivors; however, it is important to acknowledge that MBIs are not universally necessary or uniformly positive for all individuals.

Several scholars have raised concerns about potential risks or unintended consequences of MBIs in populations that are already coping effectively. Some evidence suggests that MBIs can, in a subset of individuals, heighten distress by increasing self‐focus or amplifying awareness of difficult internal states, particularly when participants have limited prior experience with contemplative practices or lack adequate psychological support [47, 48].

Another challenge to MBI implementation is ensuring consistent participation from the population of survivors. If participants have high adherence rates, the benefits abound. For example, Victorson et al. [49] found that those participants who were able to make a mindfulness practice program work with their schedules reported higher levels of self‐kindness. The literature also suggests that cancer survivors may benefit from mindfulness sessions that are delivered virtually [50]. This may mitigate some of the barriers that individuals may face when attending mindfulness‐based programs, thus potentially improving adherence.

Research shows that the psychological burdens, including, but not limited to, anxiety, depression, fear of recurrence, and distress affect TC survivors disproportionately [23, 51]. These burdens are not only persistent but are often compounded by the life stage at which TC typically occurs—young adulthood—a time characterized by identity formation, relationship development, and career establishment. MBIs have been reported to improve many of these psychological challenges, offering survivors a way to manage internal stressors through self‐regulation and present‐moment awareness [52]. Cillissen et al. [52] specifically found that among younger patients, MBIs had a greater impact on reducing psychological distress, which is notable considering the demographic distribution of TC diagnoses.

### 3.3 A Call to Action

It is well documented, as mentioned previously, that TC survivors have unique experiences with stress, coping, and HRQoL, as compared to other cancer survivors [19, 53], and as the evidence has shown, there are limited interventions to help moderate these physical and mental health concerns. These authors primarily identified physical activity trials as the primary intervention method to improve post‐treatment HRQoL given the promising outcomes reported [20, 32, 33]. Other trials that address the behavioral, social, and/or environmental factors of TC survivor HRQoL (of which there are very few) are either: (1) limited in scope due to methodological limitations, (2) their intervention is so specific that the ability for others to undertake these methods in a replication study would be difficult, (3) too complex or arduous (physically or even resourcefully speaking) for the behaviors to be sustainable among the population post‐intervention, and/or (4) lack the structural support services necessary to train/teach survivors specific therapeutic skills they can perform on their own. It is critical for the field to begin producing wellness‐promoting interventions that are sustainable, resource‐minimal, and convenient.

It appears that mindfulness‐based programming may fall into these categories and that TC survivors would greatly benefit from such services. The theoretical relevance of mindfulness‐based interventions for TC survivors is rooted in the alignment between TC‐specific stressors and core mindfulness mechanisms. Concerns related to masculinity, body image, and sexual functioning following orchiectomy may be exacerbated by self‐judgment and experiential avoidance; mindfulness practices emphasize non‐judgment and acceptance, which may reduce shame‐based appraisal and identity threat.

Fear of recurrence, a prominent and persistent concern among TC survivors, is closely linked to anticipatory anxiety and rumination. Mindfulness directly targets these processes by cultivating present‐moment awareness and reducing cognitive fixation on uncertain future outcomes. Additionally, TC survivors often report diminished perceived control in the post‐treatment period. Mindfulness practices that enhance self‐regulation and interoceptive awareness may improve self‐efficacy and psychological flexibility, allowing survivors to engage with uncertainty without heightened distress. Together, these mechanisms provide a theoretically coherent rationale for why MBIs may be particularly well‐suited to address the multidimensional psychosocial challenges characteristic of TC survivorship.

Through this review, we propose the potential benefits of MBIs in counteracting many of the disproportionate psychosocial burdens experienced by survivors of TC. MBIs are highlighted in the literature to provide benefit when incorporated on a practical basis for a multitude of cancers and various population groups, suggesting that all patients could potentially benefit from the practice, not just those experiencing mental health issues. Research has demonstrated the benefits of these programs in other populations, but additional investigation is warranted to evaluate the use of MBIs among TC survivors. Such research is necessary to determine the efficacy and effectiveness of these programs within this group, and to identify the subpopulations most likely to derive meaningful benefit.

## 4 Conclusion

MBIs have been proven to be a useful tool in mitigating the psychosocial burdens of stress, depression, and anxiety within survivors of other cancer populations [46]. MBIs can also promote self‐efficacy, which is crucial for survivors navigating a post‐treatment landscape often characterized by uncertainty and low perceived control [39, 49]. The young age of many adults diagnosed with TC may help facilitate long‐term adherence to, and efficacy of, mindfulness programs geared toward TC survivors [54, 55]. Moreover, the scalability and accessibility of MBIs, particularly when adapted for virtual delivery, make them well‐suited to overcome the logistical and structural barriers that often limit young male survivors from engaging in traditional therapy ([50]). As such, mindfulness‐based approaches represent a promising, gender‐responsive, and age‐aligned intervention for addressing the disproportionate burdens and multidimensional challenges of TC survivorship.

## Author Contributions

**Michael J. Rovito** and **Colin F. O'Mahony:** conceptualization. **Michael J. Rovito**, **Colin F. O'Mahony**, and **Kamalie Thomas:** writing – original draft preparation. **Kamalie Thomas**, **Keith Brazendale**, and **Ciaran M. Fairman:** writing – review and editing. **Michael J. Rovito:** supervision.

## Funding

This project was funded in part by the Florida Cancer Innovation Fund, Grant #25C33

## Disclosure

Precis: Testicular cancer (TC) survivors face persistent, multidimensional stressors, including anxiety, depression, and fear of recurrence, that extend well beyond physical recovery and are often unmet by existing care models. Emerging evidence suggests mindfulness‐based interventions (MBIs) offer a scalable, low‐resource, and developmentally appropriate approach to improve health‐related quality of life and psychological resilience among this uniquely vulnerable population.

## Ethics Statement

The authors have no ethical considerations to report.

## Consent

The authors have nothing to report.

## Conflicts of Interest

The authors declare no conflicts of interest.

## Data Availability

The data that support the findings of this study are available from the corresponding author upon reasonable request.
